# Unusual association of COVID-19, pulmonary tuberculosis and human immunodeficiency virus, having progressed favorably under treatment with chloroquine and rifampin

**DOI:** 10.11604/pamj.supp.2020.35.2.24952

**Published:** 2020-07-13

**Authors:** Fah Bouaré, Mehdi Laghmari, Felicité Nyafame Etouche, Badr Arjdal, Imane Saidi, Farouk Hajhouji, Houssine Ghannane, Lamyae Amro, Noura Tassi, Said Ait Benali

**Affiliations:** 1 Coronavirus Infection Unit, Department of Neurosurgery of The Arrazi Hospital, King Mohammed VI University Teaching Hospital, BP2360 Principal, Ibn Sina Avenue, Marrakesh,; 2 Coronavirus Infection Unit, Department of Infectious Diseases of the Arrazi Hospital, King Mohammed VI University Teaching Hospital, BP2360 Principal, Ibn Sina Avenue, Marrakesh,; 3 Coronavirus Infection Unit, Department of Respiratory Medicine of the Arrazi Hospital, King Mohammed VI University Teaching Hospital, BP2360 Principal, Ibn Sina Avenue, Marrakesh

**Keywords:** Coronavirus, tuberculosis, HIV, COVID-19, anti-tubercular agents, rifampin, SARS-ncov-2

## Abstract

Infection with the new coronavirus has been declared an international health emergency. Its curative treatment is unknown and is the subject of several clinical trials. In addition, the concomitant association of COVID-19 with tuberculosis and the human immunodeficiency virus, hitherto never described, is potentially fatal. We report the illustrative case of a 32-year-old patient who presented this trifecta of infections and who did well under treatment with chloroquine and anti-mycobacterial drugs. This patient arrived at the ER with respiratory discomfort that had been evolving over a month with symptoms of flu and deterioration of her general condition. A chest CT scan revealed an aspect of lung miliary tuberculosis with isolation of Koch’s bacilli in the sputum. A polymerization chain reaction (PCR) was positive for COVID-19 on a nasopharyngeal swab. HIV serology was positive. The course was marked by a spectacular clinical improvement and two negative COVID-19 PCR controls at the end of treatment (at days 9 and 10). Anti-tubercular drugs (especially, rifampin) are powerful enzyme inducers that can reduce the effectiveness of chloroquine in our patient. This therapeutic success may be linked to the effect of anti-tubercular drugs against SARS ncov-2, especially rifampin, inhibiting the formation of messenger RNAs of SARS ncov-2 or to the synergistic effect of chloroquine and rifampin. Researchers should explore the effect of these drugs on SARS ncov-2.

## Introduction

The new coronavirus disease 2019 (COVID-19) may cause severe acute respiratory syndrome (SARS) [1,2]. It is an infection affecting humans, which started in China, in the region of Wuhan, in the province of Hubei. The infection has been declared as an international health emergency [3]. Its curative treatment is unknown, and is the subject of several clinical trials on several types of molecules. We report the illustrative case of a patient who presents this rare combination of poly-infection (tuberculosis, human immunodeficiency virus and COVID-19) and who has progressed well with treatment with chloroquine and anti-tubercular drugs. In the light of this singular observation, we will propose new theories of the action of rifampin on COVID-19 and of the synergistic anti-retroviral and immunomodulatory association of the association of chloroquine with rifampin.

## Patients and Observation

**Figure 1:**
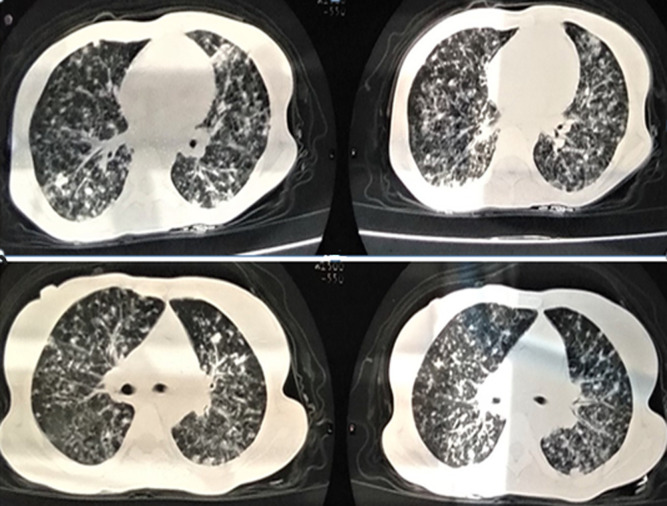
a thoracic computed tomography without injection of contrast product showing multiple micronodules, very likely in relation to a miliary tuberculosis, without typical signs of COVID-19

We report a rare case of a poly-infection (with COVID-19) with a very good course of the COVID-19 infection under chloroquine and rifampicin. The patient was a 32-year-old female, with no peculiar medical history, who presented to the ER with respiratory discomfort which had progressed for a month with influenza-like symptoms (feeling of fever, cough, headache, myalgia). She was feverish at 38.1 degrees celsius, had polypnea at 23 cycles per minute, normal heart rate at 80 beats per minute with an arterial pressure at 95/70 millimeters of mercury, her weight was 50 kilogram for a height of 1.60 meter. She had edema on her lower limbs and stage 1 gluteal ulcers. Upon suspecting COVID-19 infection in the patient, lab and imaging studies were carried out: A Polymerization Chain Reaction was positive for COVID-19 on a nasopharyngeal sample; A thoracic computed tomography (Figure 1) was able to identify multiple micronodules very likely related to a miliary tuberculosis. The bacteriological study of 3 sputum samples showed an infection with Koch´s bacillus. Human immunodeficiency virus (HIV) serology was positive with a low CD4 T lymphocytes count of 32 elements/microliter. The patient had anemia with 7.6 gram/deciliter hemoglobin, thrombocytopenia of 70,000 elements/microliter, leucopenia of 2,880 elements/microliter, hyper-ferritinemia at 8,972 nanogram/milliliter.

The diagnosis of lung miliary tuberculosis with COVID-19 infection was evoked, in a setting of retroviral immunosuppression with HIV. The patient was put on anti-tubercular treatment at the daily dose adapted to her weight (isoniazid 5 milligram/kilogram/day, rifampin 10 milligram/kilogram/day, pyrazinamide 30 milligram/kilogram/day and ethambutol 25 milligram/kilogram/day). For the treatment of COVID-19, the patient was put on chloroquine (500 milligram x 2/day) for 10 days and azithromycin (500 milligram the first day then 250 milligram/day for 7 days). The progression was marked by the obtaining of two negative PCR controls at the end of treatment (on days 9 and 10), defervescence of the fever, reduction in respiratory discomfort and cough. Currently, after a follow-up of one and a half months, she continues to improve under anti-mycobacterial treatment, with a weight of 53 kilogram and good cicatrization of the gluteal ulcers.

## Discussion

At this time of the corona virus pandemic there is no consensus on the ideal therapy for this infection [4,5]. Therapeutic trials, in the clinical trials register of the World Health Organization (WHO), have been conducted on the efficacy of certain molecules including chloroquine [6]. Chloroquine is an anti-malarial molecule which acts by the accumulation of heme of hemoglobin, which is toxic to the parasite which causes malaria [7] and an anti-rheumatic treatment [8]. It was administered to the patient at a standard dose and duration recommended for all our COVID-19 patients, because of its effectiveness on the coronavirus by inhibiting its proliferation in-vitro [9,10]. For lack of effective treatment proven by studies with a high level of evidence, chloroquine is adopted in empirical practice in a majority of centers worldwide. In addition, the treatment of tuberculosis is based on the use of anti-tubercular drugs [4,7,11]. Pulmonary tuberculosis patients benefit from an initial treatment of two months combining: isoniazid, rifampin, pyrazinamide and ethambutol [2]. These are molecules with intracellular and or extracellular actions acting on Koch´s bacillus. Rifampin is bactericidal by inhibiting RNA polymerase during the transcription of bacterial deoxyribonucleic acid (DNA) into messenger ribonucleic acid (RNA) [7].

In addition, HIV infection causes immunodepression which is a risk factor for severe forms of coronavirus infection according to the literature [4]. Anti-tubercular drugs (especially, rifampin) are powerful enzyme inducers [2,4,8] that can reduce the effectiveness of chloroquine in our patient. And immunodepression induced by HIV infection can make COVID-19 infection severe. The success of the treatment which resulted in obtaining 2 negative polymerization chain reaction COVID-19 tests, and the improvement of clinical signs, may be linked to an effect of the anti-tubercular drugs against the SARS-cov-2 infection or to the synergistic anti-retroviral effect of the chloroquine-rifampin combination. The action of rifampin, inhibiting the formation of messenger RNAs, should put researchers on the track of its anti-retroviral efficacy on COVID-19 by inhibiting or blocking the synthesis of viral proteins, as well as the track of the synergistic anti-retroviral and immunomodulatory effect of the chloroquine-rifampin combination. Large-scale, randomized studies are needed to verify these hypotheses.

## Conclusion

The new coronavirus 2019 infection is currently a real public health problem worldwide. Its treatment is not yet well codified and consensual. The action of rifampin, which inhibits the formation of messenger RNAs, transfer RNAs and ribosomal RNAs, should put researchers on the track of the antiviral efficacy of anti-tubercular drugs or of rifampin on COVID-19. And possibly, that of the synergistic chloroquine-rifampin association could be explored by large-scale, randomized studies, to verify these hypotheses.
